# Current progress in stem cell therapy for type 1 diabetes mellitus

**DOI:** 10.1186/s13287-020-01793-6

**Published:** 2020-07-08

**Authors:** Shuai Chen, Kechen Du, Chunlin Zou

**Affiliations:** Key Laboratory of Longevity and Ageing-Related Disease of Chinese Ministry of Education, Center for Translational Medicine and School of Preclinical Medicine, Guangxi Medical University, Nanning, 530021 Guangxi China

**Keywords:** Type 1 diabetes mellitus, Stem cells, Insulin-producing cells, Pancreatic islets, Transplantation

## Abstract

Type 1 diabetes mellitus (T1DM) is the most common chronic autoimmune disease in young patients and is characterized by the loss of pancreatic β cells; as a result, the body becomes insulin deficient and hyperglycemic. Administration or injection of exogenous insulin cannot mimic the endogenous insulin secreted by a healthy pancreas. Pancreas and islet transplantation have emerged as promising treatments for reconstructing the normal regulation of blood glucose in T1DM patients. However, a critical shortage of pancreases and islets derived from human organ donors, complications associated with transplantations, high cost, and limited procedural availability remain bottlenecks in the widespread application of these strategies. Attempts have been directed to accommodate the increasing population of patients with T1DM. Stem cell therapy holds great potential for curing patients with T1DM. With the advent of research on stem cell therapy for various diseases, breakthroughs in stem cell-based therapy for T1DM have been reported. However, many unsolved issues need to be addressed before stem cell therapy will be clinically feasible for diabetic patients. In this review, we discuss the current research advances in strategies to obtain insulin-producing cells (IPCs) from different precursor cells and in stem cell-based therapies for diabetes.

## Introduction

**Fig. 1:**
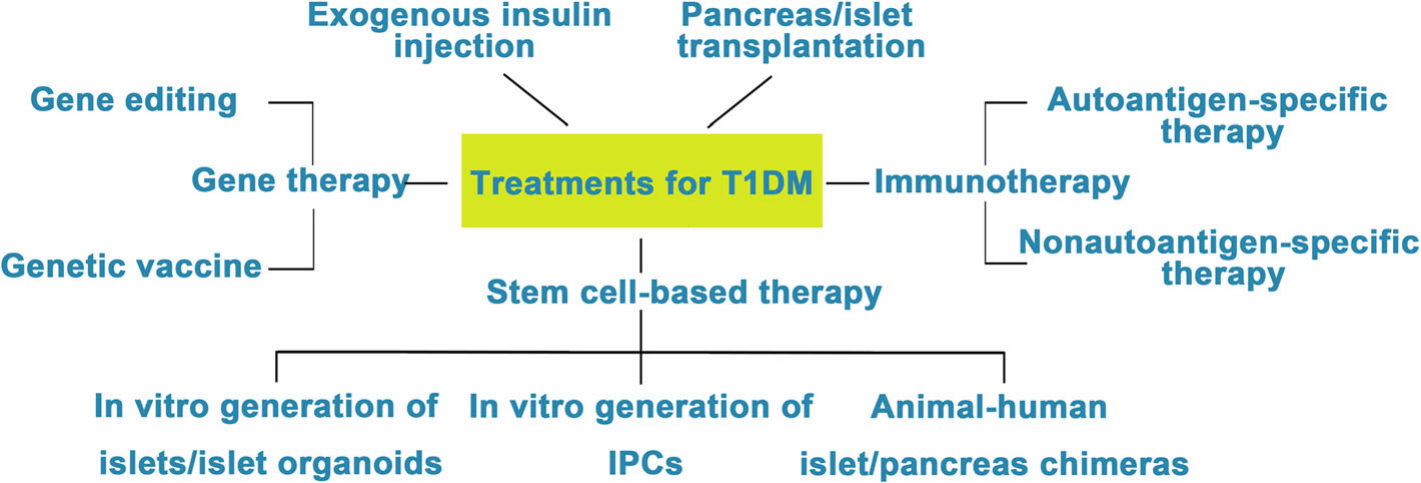
Attempts to cure T1DM. The discovery of insulin has enhanced the life span of T1DM patients, and successes in islet/pancreas transplantation have provided direct evidence for the feasibility of reestablishing β cells in vivo to treat T1DM. However, the restriction of a pancreas shortage has driven scientists to generate IPCs, and even whole pancreas, in vitro from hESCs, iPSCs, and adult stem cells. Studies focusing on the immune mechanism of T/B cell destruction in T1DM have made breakthroughs. Gene therapy has shown great promise as a potential therapeutic to treat T1DM, although its safety still needs to be confirmed in humans

Diabetes mellitus (DM) is a group of chronic metabolic disorders characterized by hyperglycemia due to insufficient secretion of insulin or insulin resistance. DM is mainly divided into four categories: type 1 diabetes mellitus (T1DM), type 2 diabetes mellitus (T2DM), gestational diabetes, and monogenic diabetes. Patients with T1DM need daily insulin injections because of the absolute insufficiency of endogenous insulin caused by autoimmune destruction of pancreatic β cells. Thus, type 1 diabetes is also known as insulin-dependent DM. Patients with type 2 diabetes may need exogenous insulin injections when oral medications cannot properly control the blood glucose levels. Diabetes without proper treatment can cause many complications. Acute complications include hypoglycemia, diabetic ketoacidosis, or hyperosmolar nonketotic coma (HHNC). Long-term complications include cardiovascular disease, diabetic nephropathy, and diabetic retinopathy [1]. Although hyperglycemia can be ameliorated by drugs or exogenous insulin administration, these treatments cannot provide physiological regulation of blood glucose. Therefore, the ideal treatment for diabetes should restore both insulin production and insulin secretion regulation by glucose in patients (Fig. 1).

Clinical pancreas or islet transplantation has been considered a feasible treatment option for T1DM patients with poor glycemic control. Dr. Richard Lillehei performed the first pancreas transplantation in 1966 [2]. Up until 2015, more than 50,000 patients (> 29,000 in the USA and > 19,000 elsewhere) worldwide had received pancreas transplantations according to the International Pancreas Transplant Registry (IPTR) [3]. Islet cell transplantation was first performed in 1974. However, efforts toward routine islet cell transplantation as a means for reversing type 1 diabetes have been hampered by limited islet availability and immune rejection. In 2000, Shapiro et al. reported that seven consecutive patients with type 1 diabetes attained sustained insulin independence after treatment with glucocorticoid-free immunosuppression combined with the infusion of adequate islet mass. Moreover, tight glycemic control and correction of glycated hemoglobin levels were observed in all seven patients. This treatment became known as the Edmonton protocol [4]. Over the past two decades, continuous improvements in islet isolation and immunosuppression have increased the efficiency of pancreatic islet transplant, and approximately 60% of patients with T1DM have achieved insulin independence 5 years after islet transplantation [3, 5–8].

However, the worldwide shortage of pancreas donors in clinical islet transplantation remains a major challenge. Intensive studies have been conducted for the generation of IPCs or islet organoids in vitro since human pluripotent stem cells (hPSCs) have been anticipated for application in regenerative medicine. The sources for the generation of IPCs or islet organoids in vitro mainly include hPSCs (human embryonic stem cells (hESCs) and human induced pluripotent stem cells (hiPSCs)), adult stem cells, and differentiated cells from mature tissues that can be transdifferentiated into IPCs. Current strategies for generating IPCs are mainly based on approaches that mimic normal pancreas development. The obtained IPCs are supposed to express specific biological markers of normal β cells that identify a terminal differentiation status, such as MAFA (a basic leucine zipper transcription factor expressed in mature β cells and absent in pancreatic progenitors and other cell types), NEUROD1 (downstream factor of NGN3 expressed in most pancreatic endocrine cells, including β cells), and PDX1/NKX 6.1 (restricted coexpression in β cells), as well as key functional features of adult β cells, including glucose-stimulated insulin secretion (GSIS) and C-peptide secretion [9–14]. In addition, after implantation into DM patients or immunodeficient diabetic animals, these in vitro-generated IPCs or islet organoids should respond to changing blood glucose and produce sufficient insulin and finally reverse hyperglycemia.

In the last two decades, many protocols have been successfully designed for the generation of IPCs or islet organoids in vitro. In this review, we summarized the research progress in the generation of IPCs and islet organoids from hPSCs and adult stem cells and the new technological advances in stem cell-based therapy for T1DM.

### Generating IPCs from embryonic stem cells (ESCs) and induced pluripotent stem cells (iPSCs)

ESCs are pluripotent cells isolated from the inner cell mass of a blastocyst, the early mammalian embryo that implants into the uterus. ESCs show the characteristics of infinite proliferative capacity and self-renewal and are able to differentiate into multiple types of adult cells in vitro [15]. iPSCs, which are reprogrammed from somatic cells, hold a similar capacity to proliferate and differentiate like ESCs. Hence, hPSCs provide a promising platform to produce in vitro insulin-secreting cells. Ethical issues in the applications of ESCs are still controversial due to their origins. In contrast, iPSCs are derived from adult somatic cells that have been reprogrammed back into an embryonic-like pluripotent state using Yamanaka factors [16, 17]. During the last two decades, numerous methods to generate IPCs from hPSCs have been reported [9–12, 18–22].

**Fig. 2:**
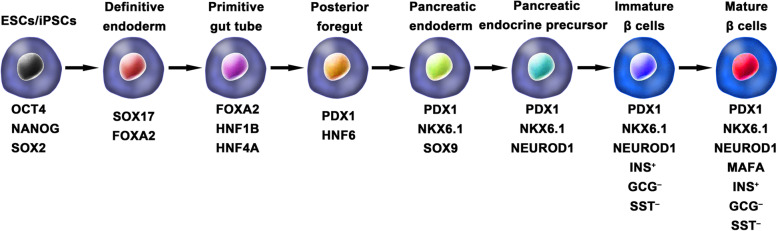
Generation of insulin-producing β cells from hPSCs. Schematic illustration of the differentiation protocol for generating insulin-producing β cells from hPSCs by mimicking the in vivo development of the embryonic pancreas. The key molecules of all key developmental stages of pancreatic islet β cells are illustrated

Ordinarily, the schemes for the generation of functional IPCs from hPSCs were based on imitating the in vivo development of the embryonic pancreas (Fig. 2). The pivotal stages of embryonic pancreas development include the development of the definitive endoderm (DE), primitive gut tube (PGT), pancreatic progenitor (PP), endocrine progenitor (EP), and hormone-expressing endocrine cells. By adding diverse cytokines (e.g., epidermal growth factor, bFGF) and signaling modulators (e.g., bone morphogenetic proteins, γ-secretase inhibitors) to each stage to activate or inhibit specific signaling pathways (e.g., Notch, Wnt) involved in the generation of adult β cells, the hPSC cell fate is manipulated into the β cell phenotype [18, 20, 23].

D’Amour et al. set up the first stepwise protocol to produce endocrine hormone-expressing cells that were able to synthesize and release multiple hormones from hESCs. However, at the final stage, the average percentage of insulin-positive cells in differentiated hES cell cultures was only 7.3%. Furthermore, these polyhormonal cells failed to respond to a high-glucose stimulus [18]. It is known that the fetal pancreas also possesses these characteristics, and previous studies demonstrated that fetal human pancreatic tissues could develop functionally after transplantation into animals [24–27]. Thus, the authors chose to determine whether these immature β cells derived from hESCs could mature into functional β cells under an in vivo environment. They generated pancreatic endoderm cells (similar to fetal 6- to 9-week pancreatic tissue) using an optimized protocol and then transplanted them into immunodeficient mice. The pancreatic endoderm cells successfully differentiated and matured into β-like cells in response to both fasting-induced hypoglycemia and glucose challenge and maintained normal glucose homeostasis for 3 months [28].

Similarly, the generation of IPCs from iPSCs is based on consecutive regulation of specific signaling pathways involved in pancreas development. Tateishi et al. first demonstrated that skin fibroblast-derived iPSCs were capable of producing islet-like clusters (ILCs) in vitro by mimicking the in vivo development of the pancreas. However, under high glucose stimulation (40 mM), the amount of C-peptide secreted by iPSC-derived ILCs and ESC-derived ILCs was only 0.3 ng/μg DNA and 0.15 ng/μg DNA, respectively [29].

Although the above studies have confirmed that hESCs and hiPSCs have the potential to differentiate into IPCs, this differentiation is done only cautiously owing to the low differentiation efficiency of protocols and the polyhormonal features of these β-like cells.

One of the breakthroughs comes from Rezania et al. in 2014, and the authors reported a more detailed protocol and generated mature and functional IPCs from hPSCs that were comparable to human β cells. The differentiation protocol was divided into 7 sequential stages, including definitive endoderm (stage 1), primitive gut hub (stage 2), posterior foregut (stage 3), pancreatic endoderm (stage 4), pancreatic endocrine precursors (stage 5), immature β cells (stage 6), and maturing β cells (stage 7). The obtained cells expressed key markers of mature β cells, such as MAFA, PDX1/NKX6.1, and INS, and showed functional similarities to human islets after transplantation in vivo. These β-like cells rapidly reversed hyperglycemia in STZ-diabetic mice by secreting C-peptide and insulin [20]. Nevertheless, the S7 (stage 7) cells were not equivalent to mature human β cells. S7 cells exhibited a very small and blunt response to high glucose stimulation, which differs from that of mature islet β cells. Moreover, a scalable suspension-based culture system developed by Paliuca et al. showed the possibility of generating large-scale stem cell-derived β cells (SC-β) [9]. Expression of NGN3 marks the initiation of endocrine differentiation. Previous studies have confirmed that inhibition of the Notch signaling pathway using γ secretase inhibitors or BMP inhibitors is essential for the induction of NGN3, followed by the addition of fibroblast growth factor 10 and keratinocyte growth factor (KGF), resulting in the robust generation of PDX1^+^ pancreatic progenitors and an increase in insulin expression in hPSC-derived progeny [9, 20]. However, Russ et al. demonstrated that the use of BMP inhibitors promoted the precocious induction of endocrine differentiation in PDX1^+^ pancreatic progenitors and that omitting addition at pancreatic specification could successfully reduce the formation of polyhormonal cells. Subsequent exposure to retinoic acid and epidermal growth factors (EGF)/KGF cocktail efficiently induced the formation of PDX1^+^/NKX6.1^+^ progenitor cells that differentiated into IPCs in vitro [10]. Recently, Yabe et al. reported that the addition of the selective glycogen synthase-kinase-3 β (GSK-3β) inhibitor (a substitute for Wnt3a; regarded as a key molecule for definitive endodermal induction from hPSCs) during definitive endodermal induction significantly decreased the death rate of endodermal cells [12, 18, 30]; further, spheroid formation of postendocrine progenitor cells rather than monolayer formation was crucial for generating IPCs from hiPSCs, which may be explained by the unique architecture of adult islets.

Among the above studies, the obtained cell population contains an average of 45% β cells, and the phenotypes of the remaining cells were unclarified. Identification of cell types that formed during differentiation is particularly important to improve the differentiated proportion of β cells. In a recent study, single-cell RNA sequencing in hPSCs undergoing in vitro β cell differentiation mapped a comprehensive description of cell production during stem-to-β cell differentiation [31]. Four distinct cell populations were isolated and identified from stem cell-derived islets, including SC-β cells, α-like polyhormonal cells, nonendocrine cells, and stem cell-derived enterochromaffin (SC-EC) cells. An in vitro study confirmed that α-like polyhormonal cells were transient toward SC-α cells and that nonendocrine cells were capable of generating exocrine cells (pancreatic acinar, mesenchymal and ductal cells). Additionally, CD49a was characterized as a surface marker of SC-β cells but not of adult islet β cells. Furthermore, SC-β cells could be purified up to 80% from SC islets using a scalable reaggregation method and magnetic sorting.

As patient-derived hiPSCs have been shown to provide tremendous advantages for studying the pathogenesis and pathophysiology of disease in vitro, studies on producing iPSCs from diabetic patients have generated great interest. Patient-specific iPSCs can overcome current obstacles in stem cell therapy, such as immune rejection and immune mismatch, and provide a platform to establish a personalized disease model to investigate pathogenic mechanisms and seek therapeutic methods for the disease. Maehr et al. successfully generated hiPSCs from skin fibroblasts of patients with T1DM (T1DM-specific iPSCs, DiPSCs). These DiPSCs resembled ESCs in the global gene expression profile and were capable of differentiating into pancreatic cell lineages, paving the path of generating T1DM SC-β cells and making autologous stem cell-derived pancreatic progeny transplantation for T1DM possible [32]. In 2015, Millman et al. confirmed that SC-β cells derived from DiPSCs functionally resembled adult islet β cells both in vivo and in vitro. GSIS tests showed that under high glucose stimulation (20 mM incubation for 30 min), T1DM and nondiabetic (ND) SC-β cells secreted 2.0 ± 0.4 and 1.9 ± 0.3 mIU of human insulin per 10^3^ cells, respectively, and both of these cells functioned similarly to adult primary islets in a previous study. After transplantation into ND immunodeficient mice, the engraft function was evaluated by serum human insulin before and 30 min after an injection of glucose. At the early time point (2 weeks after transplantation), most engrafts responded to glucose and released more insulin after glucose injection, and the ratio of insulin secretion after glucose stimulation averaged 1.4 and 1.5 for T1DM and ND SC-β cells, respectively. The effects of these engrafts on insulin secretion were observed for several months. Of note, compared to the early time point, after 12–16 weeks, the human insulin content increased approximately 1.5 times after glucose stimulation [33]. It should be acknowledged that diversities exist among T1DM patients, and a larger number of specific stem cell lines from T1DM need to be developed for future clinical use. Although DiPSCs are an alternative source for cell replacement therapy for diabetes, some T1DM-specific stem cell lines have shown low efficiency in generating PDX1^+^ pancreatic progenitors [34]. Evaluated by flow cytometry, the number of IPCs derived from ND iPSCs (25–50.5%) was comparable to that of the β cells found in human primary islets, whereas the number of IPCs differentiated from T1DM iPSC lines was much lower (15.9%) [35, 36]. Upon a strict differentiation protocol, pancreatic progenitors derived from T1DM iPSCs showed lower expression of PDX1 than ND iPSCs at a specific differentiation stage. Epigenetic changes resulting from dysmetabolism in T1DM might be responsible for the poor yield of β cells from T1DM iPSCs. Transient demethylation treatment of DE cells rescued the expression of PDX1 by inhibiting methyl group deposition on the cytosine residues of DNA and led to the differentiation of DE cells into IPCs [36]. The effect of demethylation on IPC differentiation has been shown to promote pancreatic progenitor induction rather than DE induction [37].

### Generating pancreatic progenitors from ESCs and iPSCs

Pancreatic progenitors that coexpress specific markers indispensable for inducing a β-cell fate are a crucial cell state of differentiating hPSCs into β cells in vitro. Pancreatic and duodenal homeobox 1 (PDX1) transcription factor and NK6 homeobox transcription factor-related locus 1 (NKX6.1) have been considered to be the regulatory factors of differentiating DE into pancreatic progenitors [38]. Notably, high coexpression of PDX1 and NKX6.1 in pancreatic progenitors is essential for the efficient generation of mature and functional β cells [39, 40].

Of note, the efficiency and safety of pancreatic progenitors that coexpress PDX1 and NKX6.1 for T1DM treatment are currently being evaluated in clinical trials by ViaCyte Company. Thus, elevating the production of hPSC-derived β cells, optimizing the in vitro differentiation protocols in multiple aspects, and generating a high population of PDX1^+^/NKX6.1^+^ pancreatic progenitors are needed to accelerate the clinical trial. Multiple studies have been carried out to determine the appropriate cocktail of cytokines to mimic in vivo development [41–43]. Recently, Nostro et al. demonstrated that the combination of EGF and nicotinamide induced a higher production of NKX6.1^+^ pancreatic progenitors in adherent culture [44]. Importantly, the authors focused on the temporal window of foregut differentiation into the pancreatic endoderm and confirmed that the size of the NKX6.1^+^ population decreased with extended duration. Although previous studies have shown that the maintenance of cellular aggregation during the differentiation process could significantly elevate the efficiency of pancreatic progenitors [10, 45, 46], the impact of culture condition changes that affect the physical environment of cells on pancreatic progenitor differentiation is still less studied. Memon et al. showed that the generation of PDX1^+^/NKX6.1^+^ pancreatic progenitors could be dramatically induced after dissociating and replating pancreatic endodermal cells at half density in monolayer culture [47]. Intriguingly, a novel NKX6.1^+^/PDX1^−^ cell population that holds the potential to generate functional β cells was discovered, and the cell type was confirmed to be a new type of pancreatic progenitor cell by the same team [48].

Another important issue that needs to be resolved before hPSC-derived pancreatic progenitors can be used in the clinic is how the recipient’s in vivo environment affects the maturation and differentiation of these undifferentiated cells. Although many studies have highlighted the importance of the in vivo environment in promoting islet cell differentiation, the system mechanism regulating the response of the transplanted cells to the in vivo environment has not been well studied [9, 20, 21]. Most recently, Legøy et al. confirmed that short-term exposure of encapsulated pancreatic progenitors to an in vivo environment was beneficial for cell fate determination, as revealed by increased islet proteome characteristics [49]. These effects could be partially mediated by the levels of hepatocyte nuclear factor 1-α (HNF1A) and hepatocyte nuclear factor 4-α (HNF4A) in recipients.

### Generating islet organoids/islets from ESCs and iPSCs

The pancreatic islet of Langerhans is comprised of α, β, δ, ε, and pancreatic polypeptide cells [46, 50]. Many studies have highlighted the importance of reciprocal coordination and complementary interactions of different types of islet cells for glucose hemostasis [51–54]. Thus, it may be beneficial for producing whole islets or islet organoids rather than differentiating cells into a specific type.

Organoids are defined as 3D cultures maintained in vitro that can be generated from adult tissues or hPSCs and recapitulate the in vivo morphologies, cellular architecture and organ-specific functionality of the original tissue. Kim et al. developed islet-like organoids from hPSCs that showed a glucose response in vitro and in vivo [55]. Endocrine cells (ECs) were generated from hPSCs using a multistep protocol and expressed pancreatic hormones. Notably, dissociated ECs spontaneously formed islet-like spheroids, referred to as endocrine cell clusters (ECCs), under optimal 3D culture conditions in 24 h. The diameter of the ECCs was approximately 50–150 μm and contained 5 × 10^4^ cells. ECCs consisted of several types of islet endocrine cells, apart from α cells, indicating that ECCs derived from hPSCs are partially similar to human adult islets. After high glucose stimulation (27.5 mM) for 1 h, ECCs showed increases in both insulin and C-peptide secretion, from 1.01 ± 0.22% up to 2.6 ± 0.21% and from 159.6 ± 20.01 pmol/L up to 336.3 ± 29.21 pmol/L, respectively. Additionally, ECCs exhibited intracellular Ca^2+^ oscillation under a high glucose stimulus. Furthermore, a major breakthrough was that after ECCs were implanted into STZ-induced diabetic mice, normoglycemia was rapidly achieved within 3 days. In previous studies, transplanted hPSC-derived ECs took a long period (over 40 days) to normalize the glucose level in diabetic mice [9, 10, 20, 28]. Therefore, this study suggested that it was promising to generate functional islet-like organoids from hPSCs and provided an alternative cell source for treating diabetes. Soon after that, based on a biomimetic 3D scaffold, islet organoids were successfully generated from hESCs [56]. The organoids contained all types of pancreatic cells (α, β, δ, and pancreatic polypeptide cells), specific markers of mature β cells as well as insulin secretory granules, which were characterized by a round electron-dense crystalline core surrounded by a distinctive large, clear halo. Insulin granules have been reported as an indication of mature β cells and a key participant in glucose homeostasis [36, 57]. Generally, insulin granules in adult β cells were differentiated according to the shape and density of the core. Through transmission electron microscopy, insulin granules generally possess a characteristic “halo,” which is a product of glutaraldehyde fixation that does not exist in other endocrine granules. Many studies have reported remarkable insulin granules during the differentiation of hPSCs into IPCs [9, 20]. Glucose loading experiments demonstrated that islet organoids exhibited a sharp increase in insulin secretion under high glucose conditions. Under the same glucose stimulation conditions (exposure from 5.5 mM to 25 mM), the 3D-induced cells had an insulin content that increased by seven-fold, whereas the 2D-induced cells had an insulin content that increased by 3.7-fold. These results suggested that 3D-induced IPCs are more sensitive to glucose stimulation due to their elevated maturity.

Fundamental studies of islet development during embryogenesis will promote optimization of protocols for differentiating hPSCs into 3D islet clusters or islet organoids. The traditional model of islet development is based on epithelial-mesenchymal transition (EMT) during the differentiation of pancreatic progenitors. However, this hypothesis was recently challenged by a study in which the dynamic changes in transcripts involved in islet formation were mapped [46]. Sharon et al. reported that along with EP differentiation, they maintained intact cell-to-cell adhesion and formed bud-like islet precursors (defined as peninsula-like structures) rather than undergoing EMT. Further in vitro generation of SC-β cells showed that the maintenance of cell adhesion could efficiently induce hESCs into peninsula-like structures. Importantly, these peninsula-like clusters could generate INS^+^ and GCG^+^ monohormonal cells after transplantation into SCID mice. This study provides a new framework for understanding islet embryogenesis and offers novel ideas to optimize the current protocols for the differentiation of SC-β cells.

### Generating interspecific pancreatic chimeras from pancreatic stem cells (PSCs)

Interspecific chimeras, defined as organisms with cells originating from at least two different species, are able to produce organs completely consisting of donor-origin cells. Thus, human-animal chimeras have great potential for providing immune-compatible patient-specific human organs for transplantation.

In 2010, Kobayashi et al. successfully generated a functional rat pancreas in PDX1^−/−^ (pancreatogenesis knockout) mice via interspecies blastocyst complementation [58]. The rat iPSC-derived pancreas (rat^M^ pancreas) in PDX1^−/−^ mice showed both exocrine and endocrine characteristics and expressed several pancreatic enzymes and hormones. In addition, outcomes from glucose tolerance testing (GTT) in adulthood indicated that endogenous insulin secretion was increased under high blood glucose, and glucose homeostasis was preserved. Recently, the same group reported the reverse experiment; mouse PSCs were injected into PDX1^−/−^ rat blastocysts to generate a pancreas (mouse^R^ pancreas) the size of a rat pancreas with pancreatic cells primarily originating from mouse PSCs [59]. Most importantly, the isolated islets from the mouse^R^ pancreas were subsequently injected into STZ-induced diabetic mice, and functional glucose-induced insulin secretion was successfully established in recipients for over 1 year. These data strongly supported the hypothesis that donor PSC-derived organs could be generated in a xenogeneic environment and provided the theoretical possibility of applying donor PSC-derived islets generated by animal-human interspecific blastocyst complementation in clinical trials. It is worth noting that rat^M^ pancreases were the size of a rat pancreas, rather than the size of a mouse pancreas or an intermediate size, whereas mouse^R^ pancreases were the size of a mouse pancreas. Thus, to adapt interspecific blastocyst complementation for patients, it seems necessary to generate organs in animals that are closer to humans in both size and evolutionary distance, such as sheep, pigs, and nonhuman primates (NHPs). Exogenic pancreases have been generated in vivo in transgenic cloned pigs by blastocyst complementation [60]. In this study, donor morula blastomeres derived from female cloned embryos were injected into the morula of male pancreatogenesis-disabled fetuses, and morphologically and functionally normal donor-derived pancreases were formed in adult chimeric pigs. Furthermore, PDX1^−/−^ sheep generated using CRISPR/Cas9 have been reported and can potentially serve as a host for interspecies organ generation [61]. However, blastocyst complementation has failed to generate chimeras in NHPs [62].

### Differentiation of adult stem cells into IPCs

#### The search for adult pancreatic stem cells

The adult pancreas consists of two unique parts: the exocrine pancreas and the endocrine pancreas, with unique morphology and function, respectively. The pancreas arises from two separate primordia along the dorsal and ventral surfaces of the posterior foregut. Lineage-tracing studies have demonstrated that all of the mature pancreatic cells were developed from PDX1^+^/PTF1A^+^ progenitor cells [63, 64]. However, if there are detectable pancreatic stem cells in adult animal and human pancreases, how these cells participate in the regeneration of β cells is still under debate. The hypothesis was initially supported by histological observation of neogenesis occurring in adult rodent pancreatic ducts after pancreatic duct ligation (PDL) [65]. However, genetic lineage-tracing studies indicated that there was no contribution to endocrine regeneration during the adult life or after injury, and the major mechanism was enhanced replication by only preexisting β cells [63, 66, 67]. In 2007, supporting evidence comes from a study by Xu et al., in which NGN3^+^ (the earliest islet cell-specific transcription factor) endocrine precursors appeared in the ductal lining after PDL in mice and gave rise to all types of islet cells, including glucose-responsive β cells [68]. Additionally, increased proliferation and ectopic NGN3^+^ pancreatic progenitors were reported in experiments of α-to-β-cell reprogramming [69, 70]. In conclusion, whether adult pancreatic stem cells exist in adulthood is unclear. Recent events in single-cell RNA sequencing are promising for mapping dynamic gene expression changes during the adult lifespan or after injury in animal and human pancreases, for constructing differentiation trajectories of pancreas/islet cells and for illustrating the mechanisms involved in β cell regeneration.

#### Pancreatic duct-derived stem cells

Theoretically, pancreatic duct epithelial cells possess a promising capacity for β cell generation because both originate from the same embryonic precursor [46, 71]. Budding of β cells or new islets generated from ductal epithelium occurs during pancreatic regeneration in adults and has been reported [72, 73]. Since then, studies have been designed to reprogram pancreatic ductal cells into β cells. Ramiya et al. isolated pancreatic ductal epithelial cells from prediabetic adult nonobese diabetic (NOD) mice, cultured them in vitro, and ensued the formation of ILCs that contained α, β, and δ cells. Subsequently, the blood glucose level of diabetic NOD mice was decreased from 400 to 180–220 mg/dl in 7 days [74]. Moreover, Bonner-Weir et al. demonstrated that the pancreatic ductal epithelium could expand and further differentiate into functional islet tissues in a Matrigel-based 3D culture system in vitro [75]. Further studies demonstrated that CK19^+^ nonendocrine pancreatic epithelial cells (NEPECs) can be differentiated into β cells in vitro [76].

Over the past two decades, attempts have been directed toward optimizing the protocols for generating IPCs from pancreas duct-derived stem cells. Since CA19-9 and CD133 were identified as specific membrane proteins of pancreas duct-derived stem cells, it became easier to purify these cells from the adult human pancreas [77, 78]. It has been demonstrated that diverse growth factors (e.g., bFGF, EGF, and KGF) benefit the proliferation and differentiation of human pancreatic duct-derived stem cells [74, 79]. Generally, epithelial cells show limited mitotic activity in vitro. Corritore et al. developed a differentiation protocol in which isolated human pancreatic duct cells from the pancreas were forced to undergo EMT to achieve a phenotypic change and allow them to extensively proliferate. After proliferation of these cells in vitro, pancreatic duct-derived cells differentiated into IPCs with a large array of specific marker expression and insulin secretion [78]. More recently, Zhang et al. reported that diabetic mice continuously administered gastrin and EGFs had accelerated transdifferentiation of SOX9^+^ duct cells into IPCs and consequently maintained blood glucose homeostasis [80].

#### Nestin-positive mesenchymal stem cells from islets

**Fig. 3:**
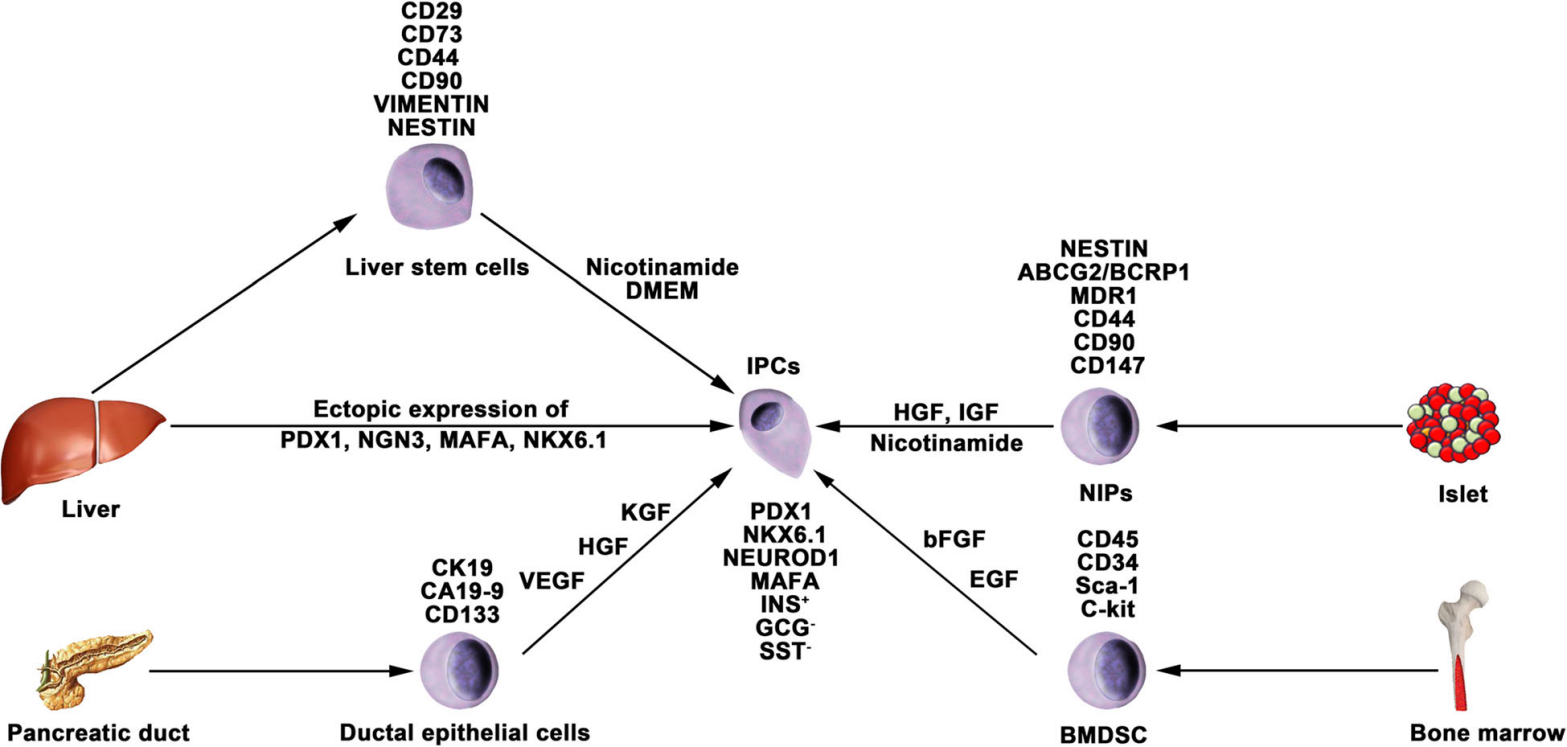
Generation of IPCs from adult stem cells. Adult pancreatic stem cells may be a potential source of IPCs. Functional IPCs have been generated from pancreatic ductal cells and NIPs isolated from adult islets. During embryogenesis, the liver and pancreas arise from common endoderm progenitors. Liver cells can transdifferentiate into IPCs by ectopic expression of pancreatic transcription factors. Additionally, a high pluripotent cell population termed HLSCs can also produce IPCs in vitro. Bone marrow-derived stem cells show the capacity to generate insulin cell clusters

Nestin is an intermediate filament protein that is specifically expressed in neuronal and muscle precursor cells [81, 82]. Recent studies have indicated that nestin-positive (nestin^+^) cells resided in pancreatic islets and could differentiate into IPCs and islet-like cell clusters (Fig. 3), and now, nestin has been accepted as a critical pancreatic progenitor marker [83, 84]. Zulewski et al. first demonstrated the existence of a distinct cell population within islets isolated from the human pancreas that express nestin, termed nestin-positive islet-derived progenitor cells (NIPs). These NIPs displayed features of stem cells and were able to generate cells with either pancreatic exocrine or endocrine phenotypes in vitro. Most importantly, the terminally differentiated cells were capable of secreting pancreatic hormones, such as insulin and glucagon [85]. Another study performed by the same group reported that NIPs also showed characteristics of bone marrow side population (SP) stem cells due to their coexpression of the ATP-binding cassette transporter ABCG2, which has been previously demonstrated to be a major component of the SP phenotype [85–87]. This was further supported by a study showing that NIPs isolated from a human fetal pancreas expressed ABCG2 and nestin [88]. Moreover, CD44, CD90, and CD147, which represent the phenotypes of bone marrow-derived mesenchymal stem cells, were also detected on NIPs. These data strongly indicated that NIPs have a high potential to become an alternative cell source for producing IPCs and islets in vitro. Huang et al. isolated and cultured NIPs from a human fetal pancreas. In this study, NIPs formed islet-like cell clusters (ICCs) in confluent cultures. Moreover, differentiation of ICCs from NIPs results in increased pancreatic islet-specific gene expression, along with a concomitant downregulation of ABCG2 and nestin. Additionally, the transplantation of ICCs reversed hyperglycemia in diabetic NOD-SCID mice [89].

The studies mentioned above about NIPs are based on rodent models. Nonhuman primate models often serve as an important bridge from laboratory research to clinical application; thus, generating pancreatic stem cells/progenitor cells from NHPs has led to great interest. Our previous study indicated that pancreatic progenitor cells existed in the adult pancreases of type 1 diabetic monkeys as well as in the pancreases of normal monkeys. The isolated pancreatic progenitor cells were able to proliferate in vitro and form ICCs in differentiation media. Furthermore, glucose-induced insulin and C-peptide secretion from the ICCs suggested that the ICCs functionally resembled primary islets [90]. In view of pathogenetic differences between STZ-induced diabetic monkeys and patients with T1DM, it still needs to be clarified whether NIPs also reside in T1DM patients.

#### Differentiation of bone marrow-derived stem cells (BMDSCs)

Several studies have reported that BMDSCs have the ability to differentiate into IPCs. Tang et al. reported that BMDSCs could spontaneously differentiate and form ICCs when continuously cultured with high glucose concentrations. The ICCs expressed multiple pancreatic lineage genes, including INS, GLUT2, glucose kinase, islet amyloid polypeptide, nestin, PDX-1, and PAX6, with β cell development. Moreover, ICCs could respond to glucose stimulation and release insulin and C-peptide in vitro, and following implantation into diabetic mice, hyperglycemia was reversed [91]. Since then, numerous studies have demonstrated the generation of IPCs from human and rat bone marrow stem cells (Fig. 3). However, the efficacy of BMDSC differentiation is low and highly variable with the current protocols. In particular, the quantity of insulin secreted by these cells was far from that secreted by adult β cells. Gabr and colleagues tested the efficiency of three differentiation protocols using immunolabeling, and the proportion of generated IPCs was modest (≈ 3%) in all protocols [92]. The expression of pancreatic-associated genes in generated IPCs was quite low compared to the expression in human islets. Optimizing differentiation protocols to upregulate the expression of specific genes by determining optimal molecules and culture conditions is crucial. Extracellular matrix proteins play a vital role in cell differentiation and proliferation. Laminin, one of the pancreatic extracellular matrices, has been confirmed to enhance the expression of insulin and promote the formation of ICCs from BMDSCs, whereas collagen type IV affects the expression of NEUROD1 and GCG [93]. Generally, differentiation of BMDSCs into IPCs is performed on nonadherent polymer surfaces and hydrogels. A recent study reported that 3D culture of BMDSCs on agar (a hydrogel-forming polysaccharide widely used in biomedical research) for 7 days followed by 2D culture of formed cellular clusters in high glucose media could enhance the production of IPCs from BMDSCs [94]. IPCs expressed INS genes at a 2215.3 ± 120.8-fold higher level than BMDSCs, whereas this fold change in previous studies was 1.2–2000-fold.

#### Differentiation of liver cells

The liver and pancreas originate from appendages of the upper primitive foregut endoderm. Later, separation of the liver and pancreas during organogenesis left both tissues with multipotent cells capable of generating both hepatic and pancreatic cell lineages. The common embryonic origin of the liver and pancreas raises the intriguing speculation that it may be possible to convert liver cells to pancreatic ECs (Fig. 3). Several studies have demonstrated that adult or fetal liver cells and biliary epithelial cells are capable of reprogramming into IPCs by inducing the expression of endocrine pancreatic-specific transcription factors [95–98]. The in vivo data showed that these hepatic cell-derived IPCs could ameliorate hyperglycemia upon implantation into diabetic mice. However, the efficiency of liver-to-pancreas reprogramming is still low, and the obtained IPCs are likely immature β-like cells. In addition, Herrera et al. isolated and characterized a population of human liver stem cells (HLSCs). HLSCs express both mesenchymal stromal cells (MSCs) and immature hepatocyte markers. In addition, HLSCs expressing nestin and vimentin are capable of differentiating into multiple cell lineages, including epithelial, endothelial, osteogenic, and islet-like structure (ILS) cells [99]. Later, Navarro-Tableros et al. confirmed that HLS-ILS cells expressed β cell transcription factors, such as NKX6.1, NKX6.3, and MAFA, and could respond to glucose loading by releasing C-peptide. Hyperglycemia was rapidly reversed in diabetic SCID mice after implantation [100]. These data suggest that HLSCs could be a novel potential resource for stem cell-based therapy for diabetes.

### Encapsulation technique for stem cell therapy for T1DM

The encapsulation technique is based on a matrix that prevents immune cells, cytokines, and antibodies from reacting to grafts while allowing nutrient, oxygen, and signaling molecule diffusion. An appropriate encapsulation device is especially crucial for T1DM to prevent an autoimmune reaction against transplanted hPSC-derived pancreatic progeny, including allogenic grafts. Criteria to evaluate an encapsulation device should take many variables into consideration, including the biocompatibility, stability and permselectivity of the membrane, interaction with the bloodstream, availability of nutrients and oxygen, among others [101–103]. Studies have been performed to detect optimal materials to improve these properties and have mainly been developed for pancreatic islet transplantation.

Alginate, a scaffolding polysaccharide produced by brown seaweeds, has been widely employed by virtue of its biocompatibility [102, 104, 105]. Alginates are linear unbranched polymers containing β-(1 → 4)-linked d-mannuronic acid (M) and α-(1 → 4)-linked l-guluronic acid (G) residues and possess eminent gel-forming properties in the presence of polyvalent cations, such as Ca^2+^ and Ba^2+^ [103, 106–108]. Earlier studies have confirmed that compared to nonencapsulated islets, encapsulated islets have significantly improved survival, long-term biocompatibility and function with the use of purified alginate [109–112]. Additionally, specific modifications to alginates trigger great interest, as they could circumvent the local immune response after transplantation of an allo- or xenograft. The incorporation of the chemokine CXCL2 with alginate microcapsules prevented allo- or xenoislet transplantation from immune reactions by establishing sustained local immune isolation [113]. Most recently, the same team confirmed that these modifications on alginates could also efficiently prolong the survival and function of hPSC-derived β cells and achieve long-term immunoprotection in immunocompetent mice with T1DM without systemic immunosuppression [114]. Of note, CXCL2 enhanced the GSIS activity of β cells, thus making it a crucial biomaterial to study for stem cell-based therapy for T1DM.

ViaCyte, leading the first and only islet cell replacement therapies derived from stem cells for diabetes, is testing for the safety and efficacy of its encapsulation devices PEC-Encap and PEC-Direct in clinical trials. The PEC-Encap is designed to fully contain hPSC-derived pancreatic progenitors in a semipermeable pouch so that vital nutrients and proteins can travel between the cells inside the device and the blood vessels, which grow along the outside of the device. In the case of PEC-Encap, the implanted cells were completely segregated from the recipients’ immune system. Another device called PEC-Direct allowed blood vessels to enter the device and directly interact with the implanted cells. Thus, immune suppression therapy was necessary for patients who received PEC-Direct, which made it suitable only for people with high-risk type 1 diabetes.

### Immune modulation in stem cell therapy for T1DM

Human ESC/iPS-derived β cells have been proposed as a potential β cell replacement source for the treatment of T1DM. However, both the alloimmune and autoimmune responses remain a major problem for the wide application of cell replacement therapies for T1DM. Although massive efforts have been made in the progress of encapsulation technology, the engraftment of transplanted hPSC-derived pancreatic progenitors or β cells still faces challenges. The engraftments will certainly be destroyed by the recipient’s immune system if the encapsulation system is eliminated. Certain modulations of these encapsulated cells to circumvent autoimmune attack seem promising. Human leukocyte antigen (HLA) mismatching is the major molecular mechanism of immune rejection in allo- or xenografts [115]. Studies have proven that elimination of HLA-A genes by zinc-finger nucleases in hematopoietic stem cells could increase donor compatibility [116, 117]. Likewise, knocking out the β2-microglobulin (B2M) gene, which abolishes all HLA class I molecules, or deleting HLA-A and HLA-B biallelically, retained one allele of HLA-C to allow the hPSC grafts to avoid T and NK cell attack [118]. Other protocols for immunosuppressive effects have been reported, such as targeted overexpression of PDL1-CTLA4Ig in β cells, which efficiently prevented the development of T1DM and allo-islet rejection, in turn promoting the survival of β cell mass [119]. Therefore, immune modulation strategies for hPSCs could be promising to overcome challenges associated with engraft rejection.

### Clinical trials in stem cell therapy for T1DM

In the last few years, controlled clinical trials have been carried out to estimate the efficiency and safety of stem cell therapy for T1DM. It has been demonstrated that MSCs can ameliorate or reverse the manifestation of diabetes in animal models of T1DM. In 2014, Carlsson et al. confirmed that MSC treatment could preserve β cell functions in new-onset T1DM patients. Twenty adult patients (aged 18–40 years) with newly diagnosed (< 3 weeks) T1DM were enrolled and randomized to MSC treatment or to the control group and followed by a 1-year follow-up examination [120]. At the end of the clinical trial, mixed-meal tolerance tests (MMTTs) revealed that both C-peptide peak values and C-peptide significantly decreased in the treatment group. Of note, MSC treatment side effects were not observed during the follow-up examination. During January 2009 and December 2010, 42 patients aged 18–40 years with a history of T1DM for ≥ 2 years and ≤ 16 years were randomized into either the stem cell transplantation (umbilical cord MSCs in combination with autologous bone marrow mononuclear cells) or standard insulin care treatment groups [121]. A 1-year follow-up examination indicated that the C-peptide increased from 6.6 to 13.6 pmol/mL/180 min in treated patients, whereas it decreased from 8.4 to 7.7 pmol/mL/180 min in control groups; insulin increased from 1477.8 to 2205.5 mmol/mL/180 min in treated patients; and it decreased from 1517.7 to 1431.7 mmol/mL/180 min in control patients. Additionally, HbA_1c_ and fasting glycemia decreased in the treated groups and increased in the control subjects. Daily insulin requirements in the treated groups also decreased compared to those of the control groups. During the follow-up period, severe hypoglycemic events reported by patients were significantly decreased. Limitations of these studies could be a small sample size and the short follow-up period. Moreover, the treated patients did not achieve complete insulin independence. Even so, these results help to improve clinical trial outcomes in future large-scale trials.

## Conclusions and perspectives

Stem cell-based therapy has been considered a promising potential therapeutic method for diabetes treatment, especially for T1DM. As mentioned in this review, major advances in research on the derivation of IPCs from hPSCs have improved our chance of reestablishing glucose-responsive insulin secretion in patients with T1DM. However, the clinical trial results of stem cell therapies for T1DM are still dissatisfactory [122], and many questions and technical hurdles still need to be solved. The major problems include the following four aspects: (1) how to generate more mature functional β-like cells in vitro from hPSCs; (2) how to improve the differentiation efficiency of IPCs from hPSCs; (3) how to protect implanted IPCs from autoimmune attack; (4) how to generate sufficient numbers of desired cell types for clinical transplantation; and (5) how to establish thorough insulin independence. Despite these obstacles, the application of stem cell-based therapy for T1DM represents the most advanced approach for curing type 1 diabetes.

## Data Availability

Not applicable.

## Abbreviations

T1DM: Type 1 diabetes mellitus
IPCs: Insulin-producing cells
DM: Diabetes mellitus
T2DM: Type 2 diabetes mellitus
HHNC: Hyperosmolar nonketotic coma
IPTR: International Pancreas Transplant Registry
hPSCs: Human pluripotent stem cells
hESCs: Human embryonic stem cells
hiPSCs: Human induced pluripotent stem cells
MAFA: MAF bZIP transcription factor A
NEUROD1: Neuronal differentiation 1
PDX1: Pancreatic and duodenal homeobox 1
NKX 6.1: NK6 homeobox transcription factor-related locus 1
GSIS: Glucose-stimulated insulin secretion
ESCs: Embryonic stem cells
iPSCs: Induced pluripotent stem cells
DE: Definitive endoderm
PGT: Primitive gut tube
PP: Pancreatic progenitor
EP: Endocrine progenitor
bFGF: Basic fibroblast growth factor
ILCs: Islet-like clusters
INS: Insulin
STZ: Streptozocin
S7: Stage 7
SC-β: Stem cell-derived β cells
NGN3: Neurogenin 3
BMP: Bone morphogenetic protein
KGF: Keratinocyte growth factor
EGF: Epidermal growth factors
GSK-3β: Glycogen synthase-kinase-3 β
SC-EC: Stem cell-derived enterochromaffin
DiPSCs: T1DM-specific iPSCs
ND: Nondiabetic
HNF1A: Hepatocyte nuclear factor 1-α
HNF4A: Hepatocyte nuclear factor 4-α
ECs: Endocrine cells
ECCs: Endocrine cell clusters
EMT: Epithelial-mesenchymal transition
GCG: Glucagon
SCID: Severe combined immunodeficiency
PSCs: Pancreatic stem cells
GTT: Glucose tolerance testing
NHPs: Nonhuman primates
PTF1A: Pancreas associated transcription factor 1a
PDL: Pancreatic duct ligation
NOD: Nonobese diabetic
NEPECs: Nonendocrine pancreatic epithelial cells
SOX9: SRY-box transcription factor 9
NIPs: Nestin-positive islet-derived progenitor cells
SP: Side population
ABCG2: ATP binding cassette subfamily G member 2
BMDSCs: Bone marrow-derived stem cells
GLUT2: Glucose transporter 2
PAX6: Paired box 6
HLSCs: Human liver stem cells
MSCs: Mesenchymal stromal cells
ILS: Islet-like structure
NKX6.3: NK6 homeobox transcription factor-related locus 3
CXCL2: C-X-C motif chemokine ligand 2
HLA: Human leukocyte antigen
HLA-A: Major histocompatibility complex, class I, A
HLA-B: Major histocompatibility complex, class I, B
HLA-C: Major histocompatibility complex, class I, C
B2M: β2-microglobulin
NK cell: Natural killer cell
PDL1-CTLA4Ig: Programmed cell death 1 ligand 1-cytotoxic T-lymphocyte antigen-4
MMTTs: Mixed-meal tolerance tests
OCT4: Octamer-binding transcription factor-4
NANOG: Nanog homeobox
SOX2: SRY-box transcription factor 2
SOX17: SRY-box transcription factor 17
FOXA2: Forkhead box A2
HNF1B: Hepatocyte nuclear factor 1-β
HNF6: Hepatocyte nuclear factor 6
SST: Somatostatin
VEGF: Vascular endothelial growth factor
HGF: Hepatocyte growth factor
IGF: Insulin-like growth factor
